# *CCR5Δ32 *variant and cardiovascular disease in patients with rheumatoid arthritis: a cohort study

**DOI:** 10.1186/ar3444

**Published:** 2011-08-16

**Authors:** Luis Rodríguez-Rodríguez, Carlos González-Juanatey, Mercedes García-Bermúdez, Tomas R Vázquez-Rodríguez, Jose A Miranda-Filloy, Benjamin Fernández-Gutiérrez, Javier Llorca, Javier Martin, Miguel A González-Gay

**Affiliations:** 1 Instituto de Parasitología y Biomedicina López-Neyra, C.S.I.C., Parque Tecnológico de Ciencias de la Salud, Avenida del Conocimiento s/n Armilla, Granada E-18100, Spain; 2 Division of Rheumatology, Hospital Clinico San Carlos, c/Profesor Martín Lagos, s/n Madrid E-28040, Spain; 3 Division of Cardiology, Hospital Xeral-Calde, c/Dr.Ochoa s/n, Lugo E-27004, Spain; 4 Division of Rheumatology, Hospital Xeral-Calde, c/Dr. Ochoa s/n, Lugo E-27004, Spain; 5 Department of Epidemiology and Computational Biology, School of Medicine, University of Cantabria, IFIMAV, and CIBER Epidemiología y Salud Pública (CIBERESP), Avenida Herrera Oria s/n, E-39011 Santander, Spain; 6 Division of Rheumatology, Hospital Universitario Marqués de Valdecilla, IFIMAV, Avenida de Valdecilla s/n, E-39008, Santander, Spain

**Keywords:** rheumatoid arthritis, atherosclerosis, cardiovascular disease, genetics, *CCR5Δ32*, rs333

## Abstract

**Introduction:**

The aim of our study was to analyze the influence of the *CCR5Δ32 *polymorphism in the risk of cardiovascular (CV) events and subclinical atherosclerosis among patients with rheumatoid arthritis (RA).

**Methods:**

A total of 645 patients fulfilling the American Rheumatism Association 1987 revised classification criteria for RA were studied. Patients were genotyped for the *CCR5 *rs333 polymorphism using predesigned TaqMan assays. Also, *HLA DRB1 *genotyping was performed using molecular-based methods. Carotid intima-media thickness, flow-mediated endothelium-dependent dilatation (FMD) and endothelium-independent vasodilatation, which were used as surrogate markers of subclinical atherosclerosis, were measured in a subgroup of patients with no clinical CV disease.

**Results:**

A lower frequency of carriers of the *CCR5Δ32 *allele among patients with CV events (3.4% versus 11.3%, *P *= 0.025, odds ratio 0.28, 95% confidence interval (95% CI) 0.06 to 0.89) was observed. However, after adjusting for gender, age at time of RA diagnosis, and the presence of shared epitope, rheumatoid factor and classic CV risk factors in the Cox regression analysis, this reduction of CV events in *CCR5Δ32 *allele carriers was slightly outside the range of significance (*P *= 0.097; hazard ratio 0.37 (95% CI 0.12 to 1.19)). Carriers of the *CCR5Δ32 *deletion also showed higher FMD values than the remaining patients (*CCR5*/*CCR5Δ32 *patients: 7.03% ± 6.61% versus *CCR5*/*CCR5 *patients: 5.51% ± 4.66%). This difference was statistically significant when analysis of covariance was performed (*P *= 0.024).

**Conclusions:**

Our results show a potential influence of the *CCR5Δ32 *deletion on the risk of CV disease among patients with RA. This may be due to a protective effect of this allelic variant against the development of vascular endothelial dysfunction.

## Introduction

CCR5 is a G protein-coupled receptor that is expressed on macrophages, monocytes, Th1 cells, immature dendritic cells, endothelial cells and vascular smooth muscle cells (VSMCs) [1-4]. The activation of this molecule through one of its ligands contributes to the survival and accumulation of macrophages [5] during inflammation, to the recruitment and activation of T cells [6] and to the activation and secretion of tissue factor [2] of VSMCs. It also participates in osteoclast formation [1]. Consistent with its roles, CCR5 is considered to play a role in both rheumatoid arthritis (RA) and atherosclerosis [7].

The *CCR5Δ32 *(dbSNP rs333) polymorphism is defined by a 32-bp deletion that leads to a truncated nonfunctional receptor [8], which is eliminated from the cell surface in homozygous individuals or its expression is reduced by 20% to 30% in heterozygous individuals [9]. Several studies have demonstrated a protective effect of the *CCR5Δ32 *allele in patients with CV disease [10,11], although others have demonstrated no association [12-14].

*CCR5Δ32 *deletion also was suggested to have a protective effect on RA susceptibility in a Spanish cohort [15]. Although other studies could not confirm a protective effect of this variant [16], a meta-analysis has suggested a protective effect [17]. When the influence of the *CCR5Δ32 *polymorphism and disease severity were analyzed, conflicting results were reported [16,18]. These contradictions may be the result of differences in study design, study power or the populations assessed; therefore, additional data will be helpful to understand the role of *CCR5 *gene polymorphisms. Taking all of these considerations together, the aim of the present study was to analyze the influence of the *CCR5Δ32 *polymorphism on the risk of CV events and subclinical atherosclerosis in patients with RA.

## Materials and methods

### Patients and study protocol

Between March 1996 and March 2008, 660 consecutive patients who fulfilled the American Rheumatism Association 1987 revised classification criteria for RA [19] were recruited from the rheumatology outpatient clinics of Hospital Xeral-Calde (Lugo, Spain) and Hospital Clínico San Carlos (Madrid, Spain). DNA samples were extracted from these patients at the time of recruitment. Between December 2009 and January 2010, patients' clinical records were examined until death, loss of follow-up or 1 December 2009. Sociodemographic and clinical data regarding clinical manifestations, classic CV risk factors and history of CV events were registered. Clinical definitions for CV events and classic CV risk factors were established as previously described [20,21]. Information on the main demographic characteristics, CV risk factors and CV events of patients in whom genotyping success was achieved (*n *= 645 (97.7%)) is shown in Table 1. Hospital Xeral-Calde and Hospital Clinico San Carlos are the referral centers for the population of each respective area. The first CV event was defined as an event (case) of CV complication diagnosed at the hospital in a patient without a history of CV disease.

**Table 1:** Demographic characteristics and genotype distribution of the patients with rheumatoid arthritis included in the study^a^

Variables	Patients (*N *= 645)
Females	484 (75.0)
Median patient age at time of disease diagnosis, years (IQR)	56 (45 to 65)
Median follow-up, years (IQR)	13 (7 to 19)
Anti-CCP-positive (*N *= 470)	283 (60.2)
Rheumatoid factor-positive (*N *= 635)	474 (74.7)
Shared epitope (*N *= 579)	366 (63.2)
Cardiovascular events	87 (13.5)
Ischemic heart disease	47 (7.3)
Cerebrovascular accidents	19 (2.9)
Heart failure	23 (3.6)
Peripheral arteriopathy	10 (1.6)
Hypertension (*N *= 640)	248 (38.8)
Diabetes mellitus (*N *= 637)	74 (11.6)
Dyslipidemia (*N *= 621)	282 (45.4)
Obesity (*N *= 610)	66 (10.8)
Smoking habit (*N *= 621)	112 (18.0)
*CCR5Δ32 *rs333	
*CCR5*/*CCR5*	579 (89.8)
*CCR5 */*CCR5Δ32*	64 (9.9)
*CCR5Δ32 */*CCR5Δ32*	2 (0.3)
*CCR5*	1,222 (94.7)
*CCR5Δ32*	68 (5.3)

^a^Anti-CCP: anticyclic citrullinated peptide antibodies; IQR: interquartile range. Values are *n *(%) except where indicated otherwise.

Endothelial dysfunction was assessed between March 2007 and September 2009 in a random subgroup of patients in the Lugo cohort with no history of CV disease. Flow-mediated endothelium-dependent vasodilatation FMD (postischemia) and endothelium-independent vasodilatation NTG (postnitroglycerin) were assessed on the basis of a brachial artery reactivity study in 127 patients as previously reported [22,23]. Intraobserver variability was 1.3% and 1.9%, respectively, based on repeat ultrasonography in 32 healthy controls. Assessment of the endothelial function of patients undergoing anti-TNF therapy was performed 24 to 48 hours before drug administration. Carotid artery intima-media thickness (IMT) was determined in 105 patients as previously reported [23,24]. The correlation coefficient was 0.98 based on repeat ultrasonography in 20 RA patients and 20 healthy controls. Participants' written consent was obtained according to the Declaration of Helsinki, and the design of the study was approved by the Ethics Committee of Galicia (Spain) and the Hospital Clinico San Carlos (Madrid).

### Genotyping

#### *CCR5 *genotyping

DNA from patients was obtained from whole peripheral blood using standard methods. Participants were genotyped to determine *CCR5 *status using TaqMan Assays-on-Demand from Applied Biosystems following the manufacturer's protocol and analyzed using the Applied Biosystems 7900 HT Fast Real-Time PCR System (Applied Biosystems, Foster City, CA, USA). The typing was successful in 645 patients (97.7%). Ten percent of the samples were regenotyped at random. We observed no differences from the results obtained before.

#### Shared epitope determination

Several *HLA-DRB1 *alleles are associated with susceptibility to RA, encoding a conserved amino acid sequence at positions 70 to 74 in the third hypervariable region, called the "shared epitope" [25]. *HLA DRB1 *typing was carried out using a reverse dot-blot kit with sequence-specific oligonucleotide probes (Dynal RELI SSO HLA-DRB1 Typing Kit; Dynal Biotech, Bromborough, UK). In our sample, 63.2% of the patients had at least one copy of the rheumatoid shared epitope, a frequency higher than that found previously in Spanish individuals without RA [26].

### Statistical analysis

Comparison of means was performed using a *t*-test. Comparison of proportions between two or more groups was carried out using a χ^2 ^test or Fisher's exact test when required. Hardy-Weinberg equilibrium (HWE) was tested in the RA patients with and without CV disease. Both groups were in HWE (*P *= 0.87 and *P *= 0.93, respectively). The study had 80% power for detecting an odds ratio (OR) ≥ 2. A Cox regression model was used to estimate the influence of the *CCR5 *polymorphism on CV disease. We used the occurrence of at least one CV event as the outcome. Survival time was defined as "age of the subjects at" or "elapsed time between RA diagnosis and" the first CV event, patient's death, loss of follow-up or 1 December 2009. Patients who died as a result of any cause other than CV events were considered not to have had CV events. Proportional hazards assumptions were tested using Schoenfeld residuals. The results are expressed as hazard ratios (HRs) with 95% confidence intervals (95% CIs) and were computed as both crude analysis and adjusted for age at RA diagnosis, gender and classic CV risk factors. The selected variables used for adjustment were selected on the basis of their association with the outcome (CV event) and the exposure (*CCR5 *genotype) and because they produced a change > 10% in the HR.

The association between *CCR5Δ32 *and carotid IMT, FMD and NTG was also tested using analysis of covariance (ANCOVA) adjusting for gender, age and duration of the disease at the time of ultrasonography as well as for the presence or absence of the shared epitope and traditional CV risk factors. This study had 80% power to detect a difference in carotid IMT of 0.1 mm or higher, a difference of 2.5% or higher in FMD and a difference of 3.5% or higher in NTG. Statistical significance was defined as *P *≤ 0.05. Calculations were performed with Stata version 10 software (StataCorp LP, College Station, TX, USA).

## Results

### Influence of the *CCR5Δ32 *polymorphism on the risk of cardiovascular events

We compared the genotypic and allelic frequencies of the *CCR5Δ32 *polymorphism between the subgroups of RA patients with and without CV events (Table 2). We found a decreased frequency of carriers of the deletion (*CCR5*/*CCR5Δ32 *+ *CCR5Δ32*/*CCR5Δ32*) among the patients with CV events (3.4% versus 11.3%, *P *= 0.025, OR 0.28 (95% CI 0.06 to 0.89)). Likewise, the *CCR5Δ32 *allele frequency was also decreased among the RA patients with CV events (1.7% versus 5.8%, *P *= 0.024, OR 0.28 (95% CI 0.06 to 0.88)).

**Table 2:** Differences between rheumatoid arthritis patients with or without cardiovascular events according to *CCR5Δ32 *polymorphism^a^

	RA patients, *n *(%)			
			
*CCR5 *genotype	With CV events	Without CV events	*P *value	OR (95% CI)
*CCR5*/*CCR5*	84 (96.6)	495 (88.7)		1
*CCR5*/*CCR5Δ32*	3 (3.4)	61 (10.9)	0.029	0.29 (0.06 to 0.92)
*CCR5Δ32*/*CCR5Δ32*	0 (0.0)	2 (0.4)	0.99	0.0 (0.0 to 31.63)
*CCR5Δ32 *carriers	3 (3.4)	63 (11.3)	0.025	0.28 (0.06 to 0.89)
Allele 2				
*CCR5*	171 (98.3)	1,051 (94.2)		1
*CCR5Δ32*	3 (1.7)	65 (5.8)	0.024	0.28 (0.06 to 0.88)

^a^CV: cardiovascular; OR (95% CI): odds ratio with 95% confidence interval; RA: rheumatoid arthritis.

We performed Cox regression analysis to account for the variation of risk of the first CV event over time according to the *CCR5*Δ32 variant, assuming a dominant model of effect (carriers versus noncarriers of the deletion) (Table 3). When age was used as a measure of survival time, to carry at least a copy of the *CCR5Δ32 *allele was not associated with a lower risk of CV disease over time, in both the crude and adjusted analyses (*P *= 0.14 and *P *= 0.14, respectively). When elapsed time from RA diagnosis was used, the reduction of CV events in carriers was slightly outside the range of significance in the crude analysis (*P *= 0.078, HR 0.35 (95% CI 0.11 to 1.12)) and in the adjusted analysis (*P *= 0.097, HR 0.37 (95% CI 0.12 to 1.19)).

**Table 3:** Cox regression model to estimate the influence of the *CCR5Δ32 *polymorphism in the risk of cardiovascular disease in patients with rheumatoid arthritis^a^

Patient group characteristics	*P *value	HR (95% CI)	*P *value^b^	HR (95% CI)^b^
Carriers vs. noncarriers^c^	0.14	0.42 (0.13 to 1.33)	0.14	0.42 (0.13 to 1.33)
Carriers vs. noncarriers^d^	0.078	0.35 (0.11 to 1.12)	0.097	0.37 (0.12 to 1.19)

^a^HR (95% CI): hazard ratio with 95% confidence interval. ^b^Analyses adjusted for gender, age at rheumatoid arthritis (RA) diagnosis, presence or absence of shared epitope, rheumatoid factor, hypertension, diabetes, dyslipidemia, obesity and smoking habit. ^c^Using as survival time the patient's age at the time of the first cardiovascular event, patient's death, loss of follow-up or 1 December 2009. ^d^Using as survival time the elapsed time between RA diagnosis and the time of the first cardiovascular event, the patient's death, loss of follow-up or 1 December 2009.

### Influence of the *CCR5Δ32 *polymorphism in subclinical atherosclerosis

Owing to the small number of homozygotes for the *CCR5Δ32 *deletion, none of those patients underwent ultrasonography for the assessment of subclinical atherosclerosis. Therefore, the comparisons were performed between heterozygote and homozygote subjects with two copies of the allele without the 32-bp deletion. In the unadjusted analysis, we did not observe a significant difference regarding carotid IMT, FMD or NTG (*P *= 0.32, *P *= 0.28 and *P *= 0.64, respectively) (Table 4). However, in the adjusted ANCOVA, we observed a significant association between being heterozygous for the *CCR5Δ32 *deletion and a higher FMD (*P *= 0.024) (Table 5). In this regard, the mean FMD percentage among heterozygotes was higher than in those without the allelic variation (7.03% ± 6.61% versus 5.51% ± 4.66%, respectively). Interestingly, the mean FMD percentage among heterozygous patients was considered normal [23].

**Table 4:** Comparison of carotid artery intima-media thickness, flow-mediated endothelium-dependent (postischemia) vasodilatation and endothelium-independent vasodilatation according to the *CCR5Δ32 *polymorphism distribution^a^

*CCR5Δ32 *polymorphism	Mean IMT, mm (SD)	*P *value	Mean FMD % (SD)	*P *value	Mean NTG % (SD)	*P *value
*CCR5*/*CCR5 *(*n *= 95)	0.73 (0.16)					
*CCR5*/*CCR5Δ32 *(*n *= 10)	0.79 (0.32)					
*CCR5Δ32*/*CCR5Δ32 *(*n *= 0)	-	0.32				
*CCR5*/*CCR5 *(*n *= 113)			5.51 (4.66)		17.2 (7.64)	
*CCR5*/*CCR5Δ32 *(*n *= 14)			7.03 (6.61)		18.21 (8.45)	
*CCR5Δ32*/*CCR5Δ32 *(*n *= 0)			-	0.28	-	0.64

^a^IMT: intima-media thickness; FMD: flow-mediated endothelium-dependent (postischemia) vasodilatation; NTG: endothelium-independent (postnitroglycerin) vasodilatation.

**Table 5:** Comparison of carotid artery intima-media thickness, flow-mediated endothelium-dependent (postischemia) vasodilatation and endothelium-independent vasodilatation according to a recessive pattern of effect of *CCR5Δ32 *polymorphism in an analysis of covariance model^a^

	*P *value		
	
*CCR5Δ32 *group	IMT	FMD	NTG
Carriers vs. noncarriers	0.77	0.024	0.11

^a^FMD: flow-mediated endothelium-dependent vasodilatation; IMT: carotid artery intima-media thickness; NTG: endothelium-independent (postnitroglycerin) vasodilatation. Analyses were adjusted for gender, age at the time of ultrasonography, follow-up time, and presence or absence of rheumatoid shared epitope, hypertension, diabetes, dyslipidemia, obesity and smoking habit.

## Discussion

This study is the first to address the role of the *CCR5Δ32 *deletion in the risk of CV disease in RA patients. We observed a lower frequency of this variant among the patients with CV complications. However, in the Cox regression model, the potential protective role of the *CCR5Δ32 *deletion was slightly outside the range of significance. Regarding the surrogate markers of subclinical atherosclerosis, we observed that RA patients with a copy of the *CCR5 *allele containing the 32-bp deletion had a higher FMD value. In fact, the mean FMD value in those patients was over the cutoff point for normal endothelial function observed in our echocardiography laboratory. These observations suggest a protective effect of the *CCR5Δ32 *deletion against the development of endothelial dysfunction, an early step in the atherogenic process, in patients with RA. Although no association of *CCR5Δ32 *deletion with carotid IMT was observed in our series, a significantly lower carotid IMT in the common carotid artery was found in individuals carrying the *CCR5Δ32 *deletion in the Bruneck study [27], which is a prospective population-based survey of the epidemiological pathogenesis of atherosclerosis. Since FMD constitutes a physiologic assessment of endothelial dysfunction and carotid IMT is an anatomic structural measure of subclinical atherosclerosis, it is logical that FMD might be a more useful diagnostic marker than carotid IMT in the early stages of the disease. In this regard, no relationship between carotid IMT and brachial artery FMD was found in middle-aged men without a history of CV disease who were considered to be at low or intermediate risk for future CV events based on current risk stratification algorithms [28]. Brachial FMD and carotid IMT values may indicate distinct and independent stages in the complex pathways leading to accelerated atherosclerosis in patients with RA. It was recently reported that, in patients with RA without CV disease, the association between FMD and carotid IMT values was observed only in patients with long disease duration [29].

As we pointed out in the Introduction, CCR5 seems to play an important role in the development of atherosclerosis. In rodent knockout models, the lack of CCR5 was associated with a reduction in plaque formation and macrophages, Th1 and smooth muscle cell accumulation, and increased expression of anti-inflammatory cytokines such as IL-10 [4,30,31]. Furthermore, studies using an antagonist of the CCR5 and CXCR3 chemokine receptors [32] or a recombinant RANTES (regulated upon activation, normal T cell expressed and secreted) receptor antagonist [33] have demonstrated an attenuation of atherogenesis in low-density lipoprotein receptor-null mice. In humans, the presence of the *CCR5Δ32 *deletion, when associated with lower or even absent expression of the CCR5 molecule on the cell surface [9], has also been associated with a lower risk of CV disease in some studies [10,11]. In the present study, we observed better endothelial function in response to ischemia among those RA patients carrying the *CCR5Δ32 *deletion. However, this fact was not associated with a strong reduction in the risk of CV disease. Since endothelial dysfunction is an early step in the atherogenic process, these observations might appear to be contradictory. However, RA is a chronic inflammatory disease, and it is well known that the persistence of chronic inflammatory burden is of major importance in the development of CV events in these patients [20]. Because of that, it is possible that a chronic inflammatory status might overcome the potential protective effect that the *CCR5Δ32 *deletion may have against the progression of the atherogenic process.

## Conclusions

In summary, our results show a potential influence of the *CCR5Δ32 *deletion on the risk of CV disease in patients with RA. This may be due to a protective effect of this allelic variant against the development of vascular endothelial dysfunction. However, further studies need to be carried out to replicate our findings and confirm the role of this molecule in the atherosclerosis disease observed in patients with RA.

## Abbreviations

ANCOVA: analysis of covariance; anti-CCP: anti-cyclic citrullinated peptide antibodies; bp: base pair; CI: confidence interval; CV: cardiovascular; FMD: endothelium-dependent flow-mediated vasodilatation (postischemia); HLA: human leukocyte antigen; HR: hazard ratio; IMT: intima-media thickness; NTG: endothelium-independent (postnitroglycerin) vasodilatation; RA: rheumatoid arthritis; RF: rheumatoid factor; TNF: tumor necrosis factor.

## Competing interests

The authors declare that they have no competing interests.

## Authors' contributions

LRR and MGB carried out genotyping, participated in the design of the study and the data analysis, and helped to draft the manuscript. CGJ performed the ultrasonographic studies, participated in the design of the study and the data analysis, and helped to draft the manuscript RPM participated in genotyping and data analysis. TRV, JAMF and LR participated in the acquisition and interpretation of data. BF was involved in the acquisition and interpretation of data and in revising it critically for important intellectual content. JM and MAGG made substantial contributions to the conception and design of the study, the acquisition of data, study coordination, helped to draft the manuscript, and gave final approval of the version to be published. All authors read and approved the final version of the manuscript for publication. MAGG and JM share senior authorship of this manuscript.
